# Solvent exposure and malignant lymphoma: a population-based case-control study in Germany

**DOI:** 10.1186/1745-6673-2-2

**Published:** 2007-04-02

**Authors:** Andreas Seidler, Matthias Möhner, Jürgen Berger, Birte Mester, Evelin Deeg, Gine Elsner, Alexandra Nieters, Nikolaus Becker

**Affiliations:** 1 Federal Institute of Occupational Safety and Health (BAuA), Berlin, Germany; 2 Department of Medical Informatics, University Medical Center Hamburg-Eppendorf, Germany; 3 Institute of Occupational Medicine, Johann Wolfgang Goethe-University, Frankfurt/Main, Germany; 4 Bremen Institute for Prevention Research and Social Medicine, Bremen, Germany; 5 German Cancer Research Center (DKFZ), Division of Cancer Epidemiology, Heidelberg, Germany

## Abstract

**Aims:**

To analyze the relationship between exposure to chlorinated and aromatic organic solvents and malignant lymphoma in a multi-centre, population-based case-control study.

**Methods:**

Male and female patients with malignant lymphoma (n = 710) between 18 and 80 years of age were prospectively recruited in six study regions in Germany (Ludwigshafen/Upper Palatinate, Heidelberg/Rhine-Neckar-County, Würzburg/Lower Frankonia, Hamburg, Bielefeld/Gütersloh, and Munich). For each newly recruited lymphoma case, a gender, region and age-matched (± 1 year of birth) population control was drawn from the population registers. In a structured personal interview, we elicited a complete occupational history, including every occupational period that lasted at least one year. On the basis of job task-specific supplementary questionnaires, a trained occupational physician assessed the exposure to chlorinated hydrocarbons (trichloroethylene, tetrachloroethylene, dichloromethane, carbon tetrachloride) and aromatic hydrocarbons (benzene, toluene, xylene, styrene). Odds ratios (OR) and 95% confidence intervals (CI) were calculated using conditional logistic regression analysis, adjusted for smoking (in pack years) and alcohol consumption. To increase the statistical power, patients with specific lymphoma subentities were additionally compared with the entire control group using unconditional logistic regression analysis.

**Results:**

We observed a statistically significant association between high exposure to chlorinated hydrocarbons and malignant lymphoma (Odds ratio = 2.1; 95% confidence interval 1.1–4.3). In the analysis of lymphoma subentities, a pronounced risk elevation was found for follicular lymphoma and marginal zone lymphoma. When specific substances were considered, the association between trichloroethylene and malignant lymphoma was of borderline statistical significance. Aromatic hydrocarbons were not significantly associated with the lymphoma diagnosis.

**Conclusion:**

In accordance with the literature, this data point to a potential etiologic role of chlorinated hydrocarbons (particularly trichloroethylene) and malignant lymphoma. Chlorinated hydrocarbons might affect specific lymphoma subentities differentially. Our study does not support a strong association between aromatic hydrocarbons (benzene, toluene, xylene, or styrene) and the diagnosis of a malignant lymphoma.

## Background

During the past decades the incidence of Non-Hodgkin lymphoma (NHL) increased in most western countries [1-4]. Only recent data indicate a potential leveling off of this trend. In Germany, NHL made up an estimated 2.7 of the male and 3.0% of the female incident cancer-cases in 2002 [5].

Several studies point to a potential etiologic role of solvents to malignant lymphoma. In the analysis of occupational groups with potential solvent exposure, Hodgkin lymphomas (HL) have been found in excess among painters [6] and workers in the chemical industry exposed to solvents [7]. Elevated Non-Hodgkin lymphoma (NHL) risks have been found among painters [8]; metal workers [9]; shoe makers and cobblers [10]; printers [11] and leather manufacturers [12]. In a previous occupation-related analysis of this study [13] based on the new WHO-classification [14,15], the following occupational groups with potential solvent exposure are positively associated with malignant lymphoma: printers; rubber and plastic product makers; shoemakers; bricklayers; carpenters; and other construction workers; maids on the level of household application; plumbers, welders, sheet metal and structural metal preparers, and erectors; metal processors; machinery fitters; and cabinet makers. However, in our study several occupations which can be expected to be prone to solvent exposure (e.g., dry-cleaners, painters) are not associated with lymphoma diagnosis.

The aim of the present multi-centre, population-based case-control study is therefore to examine the association between exposure to chlorinated hydrocarbons and lymphoma based on an in-depth expert assessment of solvent exposure.

## Methods

### Study population

The study design has been described in detail in previous publications [16,17]. Briefly, the study was conducted under the leadership of the German Cancer Research Center (DKFZ) in six defined regions in Germany: Ludwigshafen/Upper Palatinate, Heidelberg/Rhine-Neckar-County, Würzburg/Lower Frankonia, Hamburg, Bielefeld/Guetersloh, and Munich. In the mentioned study areas, all hospital and ambulatory physicians involved in the diagnosis and therapy of malignant lymphoma were asked to identify prospectively all patients between 18 and 80 years with newly diagnosed lymphoma (NHL and HL). Lymphoma patients were required to be resident in the study area and to be familiar with the German language. Of 710 participating lymphoma patients (participation rate = 87.4%), 115 suffered from HL, 554 suffered from B-NHL, 35 from T-NHL, 1 suffered from combined B-NHL and HL, and 5 from other lymphoma.

For each newly recruited lymphoma case, a gender, region and age-matched (± 1 year of birth) population control was drawn from the population registration office. Control subjects that were not familiar with the German language were excluded from the study. For each participant who had to be excluded from the study or rejected participation, the recruitment procedure was repeated. Among population controls the participation rate was 44.3%. A total of 710 case-control pairs were included in the analysis.

### Data collection

Intensively trained interviewers elicited detailed information about the medical history (including medication), lifestyle (including smoking, alcohol consumption, and leisure time activities), and occupation. The interviewers documented a complete occupational history, including every occupational period that lasted at least one year. For every job held, information was elicited about the start and the end of the job phase, about job title, industry, and specific job tasks. Study subjects having held jobs with potential relevance for lymphomagenesis (e.g., painters and lacquerers; metal workers and welders; chemical workers; shoemakers and leather workers; textile workers; dry cleaners; painters) were additionally asked to reply to job task-specific supplementary questions. For this purpose, a set of 14 job task-specific supplementary questionnaires had been developed following Bolm-Audorff et al. [18].

### Exposure assessment

A trained industrial physician (B.M.) assessed – blind to the case-control status – the intensity and frequency of exposure to specific chlorinated hydrocarbons (trichloroethylene, tetrachloroethylene, carbon tetrachlorine CTET, dichloromethane DCM) and to aromatic hydrocarbons (benzene, toluene, xylene, styrene). In a European collaborative research project with acronym EPILYMPH, this expert assessment has been coordinated by the International Agency for Research on Cancer (IARC) in Lyon. Quality assurance of expert exposure assessment included regular expert meetings and inter-rater-crosschecks of concrete assessment examples.

The intensity of exposure to specific solvents was assessed on a semiquantitative three point scale (low, medium, and high exposure), representing the absolute level of exposure in ppm. Intensity of exposure to trichloroethylene, perchloroethylene, carbon tetrachlorine, toluene, xylene, and styrene was categorized as follows: low intensity 2.5 ppm (0.5 to 5 ppm); medium intensity 25 ppm (>5 to 50 ppm); high exposure 100 ppm (>50 ppm). Intensity of exposure to dichloromethane was categorized as follows: low intensity 5 ppm (1 to 10 ppm); medium intensity 50 ppm (>10 to 100 ppm); high exposure 200 ppm (>100 ppm). Intensity of benzene exposure was categorized as follows: low intensity 2.5 ppm (0.5 to 5 ppm); medium intensity 15 ppm (>5 to 20 ppm); high intensity 50 ppm (>20 ppm).

The frequency of exposure to solvents represents the percentage of working time during which the exposure occurred (based on a 40 hours week). The frequency of exposure to specific solvents was again assessed on a semiquantitative three point scale as follows: low frequency 3% of working time (1 to 5%); medium frequency 17.5% of working time (>5 to 30%); high frequency 65% of working time (>30%). Finally, the confidence of exposure (meaning the degree of certainty according to the coder, that the worker had been exposed to the specific solvent) was assessed on a 3 point scale (possible but not probable; probable; certain).

To calculate cumulative exposure to a specific solvent [ppm*years], for every job held, the intensity of solvent was multiplied by the frequency of solvent exposure and by the corresponding duration of the job phase and summed up.

### Characteristics of cases and control subjects

The characteristics of the cases with lymphoma and control subjects are given in table 1. The mean age of cases with any lymphoma (n = 710) is 56.1 ± 16.3 years; of cases having HL (n = 116, including the person that suffered from combined B-NHL and HL), 38.8 ± 15.9 years, of cases having B-NHL (n = 554) 60.2 ± 13.6 years; and of cases having T-NHL (n = 35) 50.6 ± 17.0 years. Of the 710 case-control pairs, 55% are male and 45% are female. The average number of different occupations (held for at least 1 year) is 2.4 for lymphoma cases as well as for control subjects. The average count of different industries is 2.9 for lymphoma cases as well as for control subjects.

**Table 1:** Characteristics of cases and control subjects*

	**Control subjects** **(n = 710)**		**All lymphoma** **(n = 710)**		**Hodgkin lymphoma** **(n = 116)**		**B-Non-Hodgkin lymphoma** **(n = 554)**		**T-Non-Hodgkin lymphoma** **(n = 35)**	
	N	%	N	%	N	%	N	%	N	%
**Gender**										
Female	320	45.1	320	45.1	49	42.2	251	45.3	16	45.7
Male	390	54.9	390	54.9	67	57.8	303	54.7	19	54.3
Age at diagnosis**										
18 – 29 years	66	9.3	68	9.6	44	37.9	17	3.1	4	11.4
30 – 39 years	80	11.3	75	10.6	30	25.9	39	7.0	6	17.1
40 – 49 years	76	10.7	77	10.9	13	11.2	56	10.1	7	20.0
50 – 59 years	130	18.3	132	18.6	12	10.3	116	20.9	4	11.4
60 – 69 years	209	29.4	207	29.2	11	9.5	185	33.4	10	28.6
70 – 80 years	149	21.0	151	21.3	6	5.2	141	25.5	4	11.4
*Mean*	*56.1 ± 16.3*		*56.1 ± 16.3*		*38.8 ± 15.9*		*60.2 ± 13.6*		*50.6 ± 17.0*	
**Smoking**										
Never smoked	314	44.2	297	41.8	46	39.7	236	42.6	14	40.0
>0, <6 packyears	125	17.6	95	13.4	22	19.0	67	12.1	4	11.4
>= 6, < 22.5 packyears	137	19.3	140	19.7	24	20.7	110	19.9	5	14.3
>= 22.5 packyears	132	18.6	166	23.4	23	19.8	131	23.7	11	31.4
**Alcohol consumption**										
<2 g ethanol/day (men), <0.5 g ethanol/day (women)	126	17.7	186	26.2	47	40.9	127	22.9	11	31.4
>= 2 g ethanol/day (men), >= 0.5 g ethanol/day (women)	584	82.3	520	73.2	68	59.1	425	76.7	23	65.7

* Probands with missing information are not reported in the table

* Age of control subjects: at the time of diagnosis of the matched case

### Data analysis

At first, we analyzed the relationship between specific solvents and lymphomas as a whole (n = 710). Odds ratios (OR) and 95% confidence intervals (CI) were calculated using conditional logistic regression analysis, adjusted for smoking (in pack years) and alcohol consumption. The cumulative exposure was categorized according to the distribution among the control persons (50^th ^and 90^th ^percentile of the exposed controls). Only the results for exposure categories with at least 5 probands (cases and control subjects combined) are reported. Missing values were analyzed as a separate category (odds ratios not presented). To calculate tests for trend, the specific exposures were included as continuous variables in the logistic regression model.

Lymphomas comprise a multitude of pathogenetically different subentities with little information to what extent they are also etiologically different or share common environmental factors. Second, we therefore calculated odds ratios for the more frequent lymphoma subentities (with n > 30 cases). To increase the statistical power, patients with these lymphoma subentities were separately compared with the entire control group (n = 710) using unconditional logistic regression analysis. Covariates included in this unmatched analysis were age (as a continuous variable), sex, region, smoking, and alcohol consumption.

## Results

Table 2 presents odds ratios and 95% confidence intervals (CI) for the association between specific solvents and the entire case group (n = 710 lymphoma patients). High cumulative exposure to chlorinated hydrocarbons (>47.3 ppm*years) is statistically significantly associated with malignant lymphoma (odds ratio OR = 2.1; 95% confidence interval CI 1.1 to 4.3). When specific chlorinated solvents are analyzed, for high exposure to trichloroethylene (>35 ppm*years) an increased odds ratio of 2.1 (95% CI 1.0 to 4.8) can be seen, which is of borderline statistical significance. A non-significantly elevated lymphoma risk is evident for high exposure to tetrachloroethylene (>78.8 ppm*years) and dichloromethane (>175 ppm*years), however, numbers are small. For aromatic hydrocarbons (benzene, toluene, xylene, styrene), we find no positive association between cumulative exposure and lymphoma risk.

**Table 2:** Exposure to chlorinated and aromatic hydrocarbons and lymphoma in total (n = 710 matched pairs)

	**Cases**		**Controls**		**Adj. OR^a^**	**95% CI**
	**N**	**%**	**N**	**%**		

**CHLORINATED HYDROCARBONS**						
Chlorinated hydrocarbons in total [ppm*yrs.]						
0 ppm*yrs.	567	79.9	563	79.3	1.0	-
>0, <= 4.4 ppm*yrs.	53	7.5	74	10.4	0.7	0.5–1.0
>4.4, <= 47.3 ppm*yrs.	54	7.6	59	8.3	0.9	0.6–1.3
>47.3 ppm*yrs.	29	4.1	14	2.0	2.1	1.1–4.3
Trend test*						*P = 0.03*
Trichloroethene [ppm*yrs.]						
0 ppm*yrs.	610	85.9	602	84.4	1.0	-
>0, <= 4.4 ppm*yrs.	40	5.6	55	7.7	0.7	0.4–1.1
>4.4, <= 35 ppm*yrs.	32	4.5	44	6.2	0.7	0.5–1.2
>35 ppm*yrs.	21	3.0	9	1.3	2.1	1.0–4.8
Trend test*						*P = 0.14*
Tetrachloroethene [ppm*yrs.]						
0 ppm*yrs.	667	93.9	679	95.6	1.0	-
>0, <= 9.1 ppm*yrs.	16	2.3	16	2.3	1.1	0.5–2.3
>9.1, <= 78.8 ppm*yrs.	14	2.0	13	1.8	1.0	0.5–2.2
>78.8 ppm*yrs.	6	0.8	2	0.3	3.4	0.7–17.3
Trend test*						*P = 0.12*
Carbon tetrachlorine [ppm*yrs.]						
0 ppm*yrs.	681	95.9	696	98.0	1.0	-
>0, <= 2.3 ppm*yrs.	13	1.8	8	1.1	1.9	0.7–5.2
>2.3, <= 48.1 ppm*yrs.	8	1.1	5	0.7	2.0	0.6–6.9
>48.1 ppm*yrs.	1	0.1	1	0.1	-	-
Trend test*						*P = 0.48*
Dichloromethane [ppm*yrs.]						
0 ppm*yrs.	681	95.9	681	95.9	1.0	-
>0, <= 26.3 ppm*yrs.	8	1.1	16	2.3	0.4	0.2–1.0
>26.3, <= 175 ppm*yrs.	9	1.3	11	1.5	0.8	0.3–1.9
>175 ppm*yrs.	5	0.7	2	0.3	2.2	0.4–11.6
Trend test*						*P = 0.40*
**AROMATIC HYDROCARBONS**						
Benzene [ppm*yrs.]						
0 ppm*yrs.	591	83.2	590	83.1	1.0	
>0, <= 8.6 ppm*yrs.	53	7.5	60	8.5	0.9	0.6–1.3
>8.6, <= 130 ppm*yrs.	47	6.6	48	6.8	1.0	0.7–1.5
>130 ppm*yrs.	12	1.7	12	1.7	0.8	0.4–1.9
Trend test*						*P = 0.87*
Toluene [ppm*yrs.]						
0 ppm*yrs.	538	75.8	545	76.8	1.0	
>0, <= 3.5 ppm*yrs.	81	11.4	80	11.3	1.0	0.7–1.5
>3.5, <= 207 ppm*yrs.	70	9.9	69	9.7	1.1	0.7–1.5
>207 ppm*yrs.	14	2.0	16	2.3	0.8	0.4–1.7
Trend test*						*P = 0.74*
Xylene [ppm*yrs.]						
0 ppm*yrs.	549	77.3	552	77.7	1.0	
>0, <= 4.4 ppm*yrs.	74	10.4	80	11.3	0.9	0.7–1.3
>4.4, <= 230 ppm*yrs.	68	9.6	63	8.9	1.1	0.7–1.6
>230 ppm*yrs.	12	1.7	15	2.1	0.8	0.3–1.6
Trend test*						*P = 0.56*
Styrene [ppm*yrs.]						
0 ppm*yrs.	542	76.3	541	76.2	1.0	
>0, <= 1.5 ppm*yrs.	70	9.9	85	12.0	0.7	0.5–1.0
>1.5, <= 67.1 ppm*yrs.	79	11.1	67	9.4	1.2	0.8–1.7
>67.1 ppm*yrs.	12	1.7	17	2.4	0.6	0.3–1.4
Trend test*						*P = 0.43*

Missing values were included as a separate category (not shown)

^a ^Odds Ratio (OR) adjusted for smoking [packyears] and alcohol consumption [g per day]

* To calculate tests for trend, the exposure scores were included as *continuous *variables in the logistic regression model

Abbreviations: OR = odds ratio; CI = confidence interval; yrs. = years

In Table 3, odds ratios are reported separately for main lymphoma subentities (HL, B-cell NHL, and T-cell NHL). High exposure to chlorinated hydrocarbons remains significantly associated with B-cell NHL (OR = 2.4; 95% CI 1.2 to 4.7), but not with HL (OR = 0.5; 95% CI 0.5 to 4.6) or T-cell-NHL (OR = 1.3; 95% CI 0.1–11.4). For all main lymphoma subentities, the odds ratio for high exposure to trichloroethylene is 2.0 or more, reaching borderline statistical significance for B-cell NHL. Again the analysis of main lymphoma subentities reveals no elevated risks for the aromatic hydrocarbons benzene, toluene, xylene, or styrene.

**Table 3:** Solvent exposure and HL, B-NHL, and T-NHL (unconditional logistic regression analysis)

	**Contr.**	**HL (n = 116)**			**B-NHL (n = 554)**			**T-NHL (n = 35)**		
	**N**	**N**	**Adj. OR^a^**	**95% CI**	**N**	**Adj. OR^a^**	**95% CI**	**N**	**Adj. OR^a^**	**95% CI**

**CHLORINATED HYDROCARBONS**										
Chlorinated hydrocarbons in total [ppm*yrs.]										
0 ppm*yrs.	563	101	1.0	-	436	1.0	-	26	1.0	-
>0, <= 4.4 ppm*yrs.	74	5	0.3	0.1–0.8	45	0.8	0.5–1.2	2	0.5	0.1–2.5
>4.4, <= 47.3 ppm*yrs.	59	8	0.8	0.3–2.1	42	0.9	0.6–1.4	4	1.5	0.4–5.0
>47.3 ppm*yrs.	14	1	0.5	0.1–4.6	27	2.4	1.2–4.7	1	1.3	0.1–11.4
Trend test*			*p = 0.63 (neg.)*			*p = 0.02*			*p = 0.23*	
Trichloroethene [ppm*yrs.]										
0 ppm*yrs.	602	10.4	1.0	-	47+	1.0	-	27	1.0	-
>0, <= 4.4 ppm*yrs.	55	6	0.4	0.2–1.1	32	0.7	0.5–1.2	2	0.7	0.2–3.3
>4.4, <= 35 ppm*yrs.	44	3	0.4	0.1–1.4	27	0.8	0.5–1.3	2	1.1	0.2–5.1
>35 ppm*yrs.	9	2	2.0	0.4–10.5	17	2.3	1.0–5.3	2	4.7	0.8–26.1
Trend test*			*p = 0.97*			*p = 0.08*			*p = 0.09*	
Tetrachloroethene [ppm*yrs.]										
0 ppm*yrs.	679	111	1.0	-	521	1.0	-	30	1.0	-
>0, <= 9.1 ppm*yrs.	16	3	1.7	0.4–6.9	12	0.9	0.4–2.0	1	1.7	0.2–14.4
>9.1, <= 78.8 ppm*yrs.	13	1	0.7	0.1–6.3	12	1.0	0.5–2.3	1	1.5	0.2–12.5
>78.8 ppm*yrs.	2	-	-	-	5	3.2	0.6–16.7	1	-	-
Trend test*			*p = 0.74 (neg.)*			*p = 0.16*			*p = 0.01*	
CTET [ppm*yrs.]										
0 ppm*yrs.	696	113	1.0	-	530	1.0	-	33	1.0	-
>0, <= 2.3 ppm*yrs.	8	1	0.8	0.1–8.5	12	1.8	0.7–4.6	-	-	-
>2.3, <= 48.1 ppm*yrs.	5	1	3.7	0.4–33.6	7	1.5	0.5–4.8	-	-	-
>48.1 ppm*yrs.	1	-	-	-	1	-	-	-	-	-
Trend test*			*p = 0.86 (neg.)*			*p = 0.60*		*-*	*-*	*-*
DCM [ppm*yrs.]										
0 ppm*yrs.	681	*113*	*1.0*	*-*	531	1.0	-	32	1.0	-
>0, <= 26.3 ppm*yrs.	16	*2*	*0.7*	*0.2–3.6*	6	0.4	0.2–1.1	-	-	-
>26.3, <= 175 ppm*yrs.	11	*-*	*-*	*-*	8	0.9	0.3–2.3	1	1.2	0.1–10.9
>175 ppm*yrs.	2	*-*	*-*	*-*	5	2.7	0.5–14.5	-	-	-
Trend test*			*p = 0.24 (neg.)*			*p = 0.29*			*p = 0.5*	*(neg.)*
**AROMATIC HYDROCARBONS**										
Benzene [ppm*yrs.]										
0 ppm*yrs.	590	101	1.0	-	459	1.0	-	26	1.0	-
>0, <= 8.6 ppm*yrs.	60	9	1.0	0.4–2.3	41	0.9	0.6–1.4	3	1.2	0.3–4.4
>8.6, <= 130 ppm*yrs.	48	5	0.9	0.3–2.5	39	1.0	0.6–1.5	3	1.7	0.5–6.1
>130 ppm*yrs.	12	-	-	-	11	1.0	0.4–2.3	1	1.7	0.2–15.0
Trend test*			*P = 0.22 (neg.)*				*P = 0.50*			*P = 0.83*
Toluene [ppm*yrs.]										
0 ppm*yrs.	545	88	1.0	-	421	1.0	-	24	1.0	-
>0, <= 3.5 ppm*yrs.	80	19	1.2	0.7–2.3	56	1.0	0.7–1.5	6	1.6	0.6–4.4
>3.5, <= 207 ppm*yrs.	69	8	0.9	0.4–2.0	60	1.1	0.8–1.6	2	0.8	0.2–3.5
>207 ppm*yrs.	16	-	-		13	0.9	0.4–2.0	1	1.3	0.2–1.6
Trend test*			*P = 0.30 (neg.)*				*P = 0.43*			*P = 0.72*
Xylene [ppm*yrs.]										
0 ppm*yrs.	552	88	1.0	-	432	1.0	-	24	1.0	-
>0, <= 4.4 ppm*yrs.	80	20	1.4	0.7–2.6	48	0.8	0.5–1.2	6	1.7	0.6–4.7
>4.4, <= 230 ppm*yrs.	63	8	0.8	0.3–2.0	59	1.2	0.8–1.7	2	0.8	0.2–3.6
>230 ppm*yrs.	15	-	-	-	11	0.9	0.4–1.9	1	1.5	0.2–13.0
Trend test*			*P = 0.31 (neg.)*				*P = 0.34*			*P = 0.63*
Styrene [ppm*yrs.]										
0 ppm*yrs.	541	91	1.0	-	423	1.0	-	23	1.0	-
>0, <= 1.5 ppm*yrs.	85	11	0.4	0.2–0.8	53	0.8	0.6–1.2	6	1.3	0.5–3.6
>1.5, <= 67.1 ppm*yrs.	67	13	1.5	0.7–3.1	62	1.2	0.8–1.7	4	1.6	0.5–4.8
>67.1 ppm*yrs.	17	-	-	-	12	0.8	0.4–1.8	-	-	-
Trend test*			*P = 0.26 (neg.)*				*P = 0.18*			*P = 0.41 (neg.)*

Missing values were included as a separate category (not shown)

^a ^Odds Ratio (OR) adjusted for age, sex, region, smoking [packyears] and alcohol consumption [g per day]

* To calculate tests for trend, the exposure scores were included as *continuous *variables in the logistic regression model; (neg.) means: p for trend for a negative association

Table 4 presents odds ratios for single B-cell NHL subentities (diffuse large B-cell lymphoma DLBCL [n = 158], follicular lymphoma FL [n = 92], chronic lymphocytic leukaemia CLL [n = 104], multiple myeloma [n = 76], marginal zone lymphoma [n = 38]). Pronounced risk elevations are found for the association between high exposure to chlorinated hydrocarbons and FL (OR = 3.9; 95% CI 1.3 to 12.1) and marginal zone lymphoma (OR = 7.0; 95% CI 1.8 to 26.3). FL are statistically significantly associated with medium (but not high) exposure to toluene, xylene, and styrene, but not to benzene.

**Table 4:** Solvent exposure and B-NHL subentities (unconditional logistic regression analysis)

	**Contr.**	**DLBCL** **(n = 158)**			**FL** **(n = 92)**			**CLL** **(n = 104)**			**Multiple myeloma** **(n = 76)**			**Marginal zone lymphoma** **(n = 38)**		
	**N**	**N**	**Adj. OR^a^**	**95% CI**	**N**	**Adj. OR^a^**	**95% CI**	**N**	**Adj. OR^a^**	**95% CI**	**N**	**Adj. OR^a^**	**95% CI**	**N**	**Adj. OR^a^**	**95% CI**

**CHLORINATED HYDROCARBONS**																
Chlorinated hydrocarbons in total [ppm*yrs.]																
0 ppm*yrs.	563	132	1.0	-	70	1.0	-	78	1.0	-	62	1.0	-	27	1.0	-
>0, <= 4.4 ppm*yrs.	74	10	0.6	0.3–1.2	9	1.4	0.6–3.1	11	0.9	0.4–1.9	3	0.4	0.1–1.3	4	1.6	0.5–5.1
>4.4, <= 47.3 ppm*yrs.	59	9	0.7	0.3–1.5	8	1.7	0.7–4.0	9	0.7	0.3–1.6	8	1.0	0.4–2.4	3	1.4	0.4–5.3
>47.3 ppm*yrs.	14	5	1.8	0.6–5.3	5	3.9	1.3–12.1	6	1,8	0.6–4.9	2	0.9	0.2–4.4	4	7.0	1.8–26.3
Trend test*				*P = 0.02*			*P = 0.04*			*P = 0.88*			*P = 0.15*			*P = 0.008*
Trichloroethene [ppm*yrs.]																
0 ppm*yrs.	602	139	1.0	-	79	1.0	-	86	1.0	-	65	1.0	-	32	1.0	-
>0, <= 4.4 ppm*yrs.	55	6	0.5	0.2–1.2	7	1.3	0.5–3.2	10	1.1	0.5–2.4	3	0.5	0.2–1.9	2	0.9	1.2–4.3
>4.4, <= 35 ppm*yrs.	44	7	0.8	0.3–1.8	3	0.7	0.2–2.6	6	0.7	0.3–1.7	6	1.0	0.4–2.7	2	4.2	0.8–23.9
>35 ppm*yrs.	9	4	2.6	0.7–3.0	3	3.2	0.8–12.9	2	0.9	0.2–4.5	1	0.7	0.1–5.5	2	4.2	0.8–23.9
Trend test*				*P = 0.03*			*P = 0.16*			*P = 0.46 (neg.)*			*P = 0.43 (neg.)*			*P = 0.15*
Tetrachloroethene [ppm*yrs.]																
0 ppm*yrs.	679	146	1.0	-	90	1.0	-	101	1.0	-	33	1.0	-		1.0	-
>0, <= 9.1 ppm*yrs.	16	3	0.9	0.3–3.9	2	1.2	0.3–5.5	1	-	-	3	1.8	0.5–6.7	1	-	-
>9.1, <= 78.8 ppm*yrs.	13	6	2.1	0.8–5.9	-			2	0.6	0.1–2.8	-	-	-	3	4.2	1.02–17.5
>78.8 ppm*yrs.	2	1	2.3	0.2–26.0	-			-	-	-	-	-	-	1	-	-
Trend test*				*P = 0.19*			*P = 0.43 (neg.)*			*P = 0.6*			*P = 0.34 (neg.)*			*P = 0.10*
CTET [ppm*yrs.]																
0 ppm*yrs.	696	153	1.0	-	87	1.0	-	98	1.0	-	72	1.0	-	37	1.0	-
>0, <= 2.3 ppm*yrs.	8	1	0.7	0.1–6.1	4	5.0	1.4–18.3	5	2.7	1.8–8.9	-	-	-	-	-	-
>2.3, <= 48.1 ppm*yrs.	5	2	1.6	0.3–8.9	1	2.1	0.2–19.4	-	-	-	3	4.5	0.57–20.9	1	3.6	0.4–34.6
>48.1 ppm*yrs.	1	-	-	-	-	-	-	-	-	-	-	-	-	-	-	-
Trend test*				*P = 0.75*			*P = 0.83*			*P = 0.38*			*P = 0.83*			*P = 0.98*
DCM [ppm*yrs.]																
0 ppm*yrs.	681	*150*	1.0	-	88	1.0	-	100	1.0	-	74	1.0	-	36	1.0	-
>0, <= 26.3 ppm*yrs.	16	*3*	0.8	0.2–2.9	1	0.5	0.1–4.3	1	0.3	0.04–2.4	-	-	-	1	1.5	0.2–11.9
>26.3, <= 175 ppm*yrs.	11	*2*	0.9	0.2–4.2	2	1.5	0.3–7.4	2	0.8	0.2–3.9	-	-	-	-	-	-
>175 ppm*yrs.	2	*1*	-	-	1	-	-	1	-	-	1	-	-	1	-	-
Trend test*				*P = 0.45*			*P = 0.20*			*P = 0.78*			*P = 0.19*			*P = 0.10*
**AROMATIC HYDROCARBONS**																
Benzene [ppm*yrs.]																
0 ppm*yrs.	590	136	1.0	-	73	1.0	-	80	1.0	-	62	1.0	-	32	1.0	-
>0, <= 8.6 ppm*yrs.	60	9	0.7	0.3–1.4	10	1.8	0.8–3.8	8	0.8	0.4–1.8	6	1.0	0.4–2.4	2	0.7	0.2–3.0
>8.6, <= 130 ppm*yrs.	48	10	0.9	0.4–1.9	7	1.5	0.6–3.5	14	1.6	0.8–3.2	4	0.7	0.2–2.0	3	1.1	0.3–3.8
>130 ppm*yrs.	12	1	0.3	0.04–2.6	2	1.3	0.3–6.4	2	0.7	0.1–3.1	3	1.8	0.5–6.8	1	1.4	0.2–11.8
Trend test*				*P = 0.52 (neg.)*			*P = 0.51*			*P = 0.48*			*P = 0.54*			*P = 0.32*
Toluene [ppm*yrs.]																
0 ppm*yrs.	545	122	1.0	-	66	1.0	-	76	1.0	-	59	1.0	-	27	1.0	-
>0, <= 3.5 ppm*yrs.	80	18	1.1	0.6–2.0	8	1.1	0.5–2.4	11	0.9	0.4–1.8	6	0.8	0.3–1.9	4	1.0	0.3–3.3
>3.5, <= 207 ppm*yrs.	69	14	1.0	0.5–1.9	16	2.6	1.4–5.1	15	1.3	0.7–2.4	7	0.9	0.4–2.0	6	1.9	0.7–5.1
>207 ppm*yrs.	16	2	0.6	0.1–2.5	2	1.1	0.2–5.3	2	0.5	0.1–2.4	3	1.4	0.4–5.2	1	1.1	0.1–9.5
Trend test*				*P = 0.87*			*P = 0.48*			*P = 0.48*			*P = 0.48*			*P = 0.48*
Xylene [ppm*yrs.]																
0 ppm*yrs.	552	127	1.0	-	66	1.0	-	79	1.0	-	59	1.0	-	27	1.0	-
>0, <= 4.4 ppm*yrs.	80	13	0.8	0.4–1.5	7	1.0	0.4–2.3	9	0.7	0.3–1.5	6	0.8	0.3–1.9	5	1.4	0.5–4.0
>4.4, <= 230 ppm*yrs.	63	14	1.1	0.6–2.0	17	3.0	1.6–5.8	15	1.3	0.7–2.5	7	0.9	0.4–2.2	5	1.8	0.6–4.9
>230 ppm*yrs.	15	2	0.6	0.1–2.6	2	1.3	0.3–6.2	1	0.3	0.04–2.2	3	1.5	0.4–5.8	1	1.3	0.2–10.5
Trend test*				*P = 0.95*			*P = 0.33*			*P = 0.47*			*P = 0.36*			*P = 0.40*
Styrene [ppm*yrs.]																
0 ppm*yrs.	541	117	1.0	-	60	1.0	-	81	1.0	-	60	1.0	-	31	1.0	-
>0, <= 1.5 ppm*yrs.	85	15	0.8	0.4–1.5	12	1.1	0.5–2.1	10	1.0	0.5–2.2	6	0.8	0.3–1.9	4	1.0	0.3–3.0
>1.5, <= 67.1 ppm*yrs.	67	19	1.3	0.7–2.3	17	2.2	1.2–4.0	11	1.1	0.5–2.2	8	1.0	0.5–2.4	3	0.8	0.2–2.6
>67.1 ppm*yrs.	17	5	1.5	0.5–4.4	3	1.6	0.5–6.0	2	0.5	0.2–2.3	1	0.5	0.1–3.8	-	-	-
Trend test*				*P = 0.03*			*P = 0.20*			*P = 0.37*			*P = 0.85*			*P = 0.28*

Missing values were included as a separate category (not shown)

^a ^Odds Ratio (OR) adjusted for age, sex, region, smoking [packyears] and alcohol consumption [g per day]

* To calculate tests for trend, the exposure scores were included as *continuous *variables in the logistic regression model

Abbreviations: OR = odds ratio; CI = confidence interval; yrs. = years

## Discussion

In this study, we observed a statistically significant association between high exposure to chlorinated hydrocarbons – particularly trichloroethylene – and malignant lymphoma. In the analysis of lymphoma subentities, a pronounced risk elevation was found for follicular lymphoma and marginal zone lymphoma. Among the chlorinated hydrocarbons investigated, trichloroethylene was the solvent with the highest exposure prevalence among the control subjects: 15.2% of the control subjects were ever exposed to trichloroethylene, 20.7% were ever exposed to any chlorinated hydrocarbons, 49.0% were ever exposed to any aromatic hydrocarbons. However, the proportion of control persons ever exposed to solvents decreased to 7.9% (trichloroethylene), 10.6 (any aromatic hydrocarbons), resp. 24.9% (any aromatic solvents), when only persons with "certain" trichloroethylene exposure were regarded as exposed. Therefore, the exposure assessment might be regarded as rather sensitive, but less specific, introducing possible non-differential misclassification bias. When in an additional analysis, solely "certain" exposures were considered (regarding persons with possible and probable exposure as unexposed), odds ratios were attenuated (results not shown).

In 1995 the International Agency for Research on Cancer (IARC) classified trichloroethylene as a probable human carcinogen (Group 2A); the lymphatic system was regarded as a target for trichloroethylene toxicity [19]. Several subsequent incidence-based cohort studies supported this classification [20-22]. A recent meta-analysis of 14 occupational cohort and four case-control studies [23] reveals a modest positive association between trichloroethylene and NHL in a specific trichloroethylene-exposed sub-cohort analysis (RR = 1.6; 95% CI 1.2 to 2.1). However, the authors concluded that there is insufficient evidence for a causal link between trichloroethylene exposure and NHL. This conclusion was mainly based on the lack of a clear dose-response relationship in the reviewed studies. In our study, despite of the potential exposure misclassification, an elevated lymphoma risk can be seen in the highest trichloroethylene exposure group (table 2). When we restricted our analysis to trichloroethylene exposure which had occurred 10 or more years prior to diagnosis, this led to a slight increase in lymphoma risk (OR = 2.2; 95% CI 1.0–4.9; the complete results of this lag-time analysis are available by the authors).

Tetrachloroethylene (synonym perchloroethylene PCE), has also been used as an industrial solvent for several decades. Exposure to PCE is widely prevalent especially in dry-cleaning companies, where it was the dominant solvent since the seventies. In 1995 the IARC concluded from a limited database that exposure to PCE leads to an increased NHL risk [19]. Meanwhile conducted cohort studies with an extended follow-up period [24] and a recent case-control study in the Nordic countries [25] could not reproduce an increased NHL risk of PCE-exposed persons. In our study only about 4% of the control subjects were classified as PCE-exposed (including persons with "probable", but not "certain" PCE exposure), therefore the power of our study is limited to detect an increased lymphoma risk among PCE-exposed persons. We could find a (non-significantly) elevated lymphoma risk only for the highest PCE exposure category. It should be taken into account that most subjects exposed to PCE in our study were also exposed to trichloroethylene (76%). Therefore, the increased OR for highly PCE-exposed persons might possibly just reflect the increased OR for trichloroethylene co-exposure. This explanation is supported by a relatively high correlation between the estimated cumulative exposure to PCE and trichloroethylene (Pearson correlation coefficient = 0.42).

Only 2% of the control subjects are exposed to carbon tetrachloride according to expert assessment, leading as well to a limited power. In the early 20^th ^century, carbon tetrachloride was widely used as a dry cleaning solvent, as a refrigerant, and in fire extinguishers; later, it was used as a grain fumigant. The use of carbon tetrachloride is clearly declining since the seventies. In our study, we found an OR of about 2 among carbon tetrachloride-exposed persons, which was of borderline statistical significance. This is in accordance with the results of a Canadian case-control study, including 517 cases and 1,506 control subjects [26]. In a more recent study among nearly 5,000 Finnish laboratory workers seven NHL cases and three HL cases were found, yielding a SIR for NHL and HL of 1.54 [27]. Although it was a small study, it is noteworthy that four of the seven NHL cases were potentially exposed to carbon tetrachloride (assessed by usage of the Finnish Register of Workers Exposed to Carcinogens).

Dichloromethane (DCM), also known as methylene chloride, is mainly used as a paint remover but is also applied as a solvent and cleaning agent in a variety of industries. The few epidemiological studies investigating occupational exposures to DCM do not report increased lymphoma risks [28-30]. As in previous studies, the prevalence of DCM exposure was also low in our study (about 4% of the control subjects were ever DCM-exposed). We could observe a non-significantly elevated risk in the highest DCM exposure group (5 cases vs. 2 controls); the comparison of ever vs. never DCM-exposed persons yielded an odds ratio below one. Because of the low DCM exposure prevalence, our study power might have been insufficient to detect a slightly elevated lymphoma risk.

There is a sufficient body of evidence concerning the leukaemogenicity of benzene. Recent results in the fields of toxicology and molecular oncology have shown that not only precursor cells in bone marrow but even peripheral lymphatic cells are targeted by the genotoxic metabolites of benzene in humans in vivo, thus making it probable that all kinds of lymphoma may be induced by the compound [31-33]. However, the majority of recent cohort studies on occupational exposure to benzene failed to demonstrate an increased lymphoma risk [34-36]. One large-scale cohort study among Chinese employees [37] reports an elevated NHL risk. A difficulty in most of the cohort studies is the definition of a really null-exposed reference group. We believe that population-based case-control studies – despite their difficulty to adequately estimate exposures retrospectively – have the advantage of a relatively "undiluted" reference group. In our case-control study, 17% of cases as well as control subjects were ever exposed to benzene. Neither the comparison of ever versus never exposed (results not shown) nor the analysis of categorized cumulative benzene exposure led to increased lymphoma risks. This result is in accordance with a recently published review [38]. Concerning NHL subentities, a case-control study nested within a large cohort study among Australian petroleum workers [39] points to a dose-response relationship between benzene exposure and CLL: The OR is 2.8 (95% CI 0.4–18.1) for >4–8 vs. ≤ 4 ppm*years and 4.5 (95% CI 0.9–22.9) for >8 vs. ≤ 4 ppm*years. In our study, the CLL risk was elevated to 1.6 (95% CI 0.8–3.2) among persons exposed to >8.6 to 130 ppm*years; however, in the highest benzene exposure category (>130 ppm*years), the OR decreased to 0.7 (95% CI 0.1–3.1; n = 2 cases and 11 control subjects). Given the low numbers and the methodological difficulties of a valid retrospective exposure assessment, these results do not preclude an association between benzene exposure and specific lymphoma entities.

Toluene and xylene are the aromatic hydrocarbons with the highest exposure prevalence in our cohort; roughly a quarter of the control subjects were ever exposed to toluene resp. xylene (including rather uncertain exposures). Toluene and xylene exposure are highly inter-correlated; furthermore, exposure to both substances is highly correlated with benzene exposure (Pearson correlation coefficient between 0.94 and 0.97). Therefore, we are virtually unable to distinguish between the effects of benzene, toluene, and xylene (BTX exposure). However, we could not find any increased risks in the separate analysis of the mentioned substances as well as in the analysis of BTX exposure as a whole (results not shown). In a recently published large case-control study in Italy, Miligi and colleagues [40] found significantly elevated NHL risks among individual with medium or high benzene, toluene, or xylene exposure (OR between 1.6 and 1.8). In the mentioned case-control study, subjects with very low or low benzene, toluene, or styrene exposure show a significantly decreased NHL risk. As in our study, benzene, toluene and xylene exposure was highly correlated in the mentioned Italian study. In comparison with the Italian study, the ability of our study is more limited to detect a slightly increased lymphoma risk: If, for example, the prevalence of benzene exposure >8.6 ppm-years among the control subjects (8.5%) was equal to the true prevalence, the power of our study would have been only 69% to detect an odds ratio of 1.6 (as reported in the Italian study). Mainly because of the limited study power, we cannot exclude an etiologic relevance of BTX exposure on lymphoma. On the basis of these and the Italian data [40], a strong effect of low-dose BTX exposure on the development of lymphoma is considered as rather unlikely.

Styrene, the fourth aromatic hydrocarbon investigated in our study, is widely used in lamination of reinforced plastics, in the production of – inter alia – rubber, plastic, insulation, and fibreglass. The exposure prevalence in control subjects (23.8%) was comparable with the exposure prevalence of other aromatic hydrocarbons. The correlation between styrene and BTX solvents (Pearson correlation coefficient = 0.25) was considerably lower than the correlation within the BTX group. Our results do not support the hypothesis of a dose-response relationship between styrene and lymphoma risk.

Strengths of our study include the expert-based calculation of cumulative solvent exposure during the entire work time and adjustment for several potential confounders. The exposure assessment was conducted blind for the case-control study; we therefore regard a differential exposure misclassification as rather improbable. However, limitations of the present analysis should be considered when interpreting the results. Retrospective exposure assessment on semi-quantitative intensity and frequency scales always implies non-differential misclassification which tends to bias the effect estimate to the null. When only definite exposures were taken into consideration, specifity of the exposure assessment was expected to increase (at the cost of decreasing sensitivity): However, when only definite exposures were regarded, we did not find any risk elevations.

## Conclusion

To conclude, our study adds further evidence to the potential relevance of trichloroethylene exposure – possibly also of exposure to other chlorinated hydrocarbons – on the etiology of lymphomas. The risk for specific lymphoma subentities might differ from the overall risk for malignant lymphoma: in our study, particularly pronounced risk elevations are found for the association between high exposure to chlorinated hydrocarbons and follicular NHL as well as marginal zone NHL.

## Competing interests

The author(s) declare that they have no competing interests.

## Authors' contributions

AS participated in the design of the study, drafted the manuscript and performed the statistical analysis. MM participated in the statistical analysis and in drafting the manuscript. JB participated in the coordination of the data collection and in the critical revision of the study. BM and GE participated in the exposure assessment and in the critical revision of the manuscript. ES is the data manager of the German lymphoma study and participated in the statistical analysis and in the critical revision of this manuscript. AN and NB who is the PI prepared, designed and coordinated the study and helped to draft the manuscript. All authors read and approved the final manuscript.
